# Porcelain

**Published:** 1903-05

**Authors:** Herbert Locke Wheeler

**Affiliations:** New York


### International Dental Journal.

Vol. XXIV. May, 1903. No. 5.

### Original Communications.[1]

### PORCELAIN.[2]

BY HERBERT LOCKE WHEELER, D.D.S., NEW YORK.

The origin of this substance is lost in the hopeless obscurity of the past. It is extremely ancient. So far as we know, it was first produced in China. Writers differ as to the date when it was first known. A missionary who lived long in China says it was known in the reign of Yao and Chuen, two emperors who ruled about two thousand six hundred years before Christ, according to the native historians. Some place its discovery only two or three centuries before Christ. However this may be, it is quite certain that the art had risen to a high degree of perfection by the tenth and twelfth centuries A.D., both in China and Persia. Near Nankin stands a tower of nine stories, three hundred feet high, which is entirely covered with a fine quality of porcelain; this was built about 1277. The Chinese have been very reluctant to give informa-

[1] The editor and publishers are not responsible for the views of authors of papers published in this department, nor for any claim to novelty, or otherwise, that may be made by them. No papers will be received for this department that have appeared in any other journal published in the country.

[2] Read before The New York Institute of Stomatology, February 3, 1903.

tion concerning processes used by them in making porcelain, but with the assistance of modern science its composition has become an open book. The Portuguese traders were the first to introduce china-ware into Europe, and this ware was fairly well imitated in France about 1695. This was an artificial material, and was called tender porcelain, as it scratched easily. The first real porcelain produced in Europe was made by Böttger, a German, at Meissen, near Dresden, in 1703, and here he established the first porcelain manufactory in 1709.

All ceramics are composed of essentially the same elements, from a Sèvres vase to a drain-pipe, the difference being entirely in the proportion of the different ingredients. Porcelain, which is the highest product of the ceramic art, is a hard, vitreous, translucent substance produced by the fusing of several minerals in connection with clay. The materials used are kaolin, silica, felspar, and often is added potash, soda, or lime, to increase the alkaline flux of the felspar. Glass is also a compound of silica with potash, but differs from porcelain in that all its component parts, broadly speaking, are fusible, making a chemical mixture, while porcelain contains infusible elements held together by the lower fusing enamel; this is a mechanical mixture.

An excess of alkaline fluid may dissolve the intractable parts of a porcelain body—that is, the silica—during the fusing process, thus forming a soluble salt; this may explain the tendency of low-fusing porcelain, so called, to disintegrate in the mouth.

The material in all ceramics can be put under three headings: First, clays and marls; the former is kaolin, the latter calcium carbonate. Second, hardening or aplastic materials,—flint, sand, and quartz; these are all a double oxide of silica, and are infusible except under the oxyhydrogen blow-pipe. Third, fusible materials, —felspar, an aluminum silicate containing potash or soda, and sometimes lime and magnesia; bone or fern ash, which are calcium and potassium carbonate, the fern ash being much used by the Chinese; calcium, barytes, and artificial frits.

The quality of porcelain produced depends upon the skill in choosing materials and proportions so as to produce the results required. The element of time required for heating and cooling must also be taken into account. The variation in density which porcelain undergoes in fusing is not thoroughly understood. It appears to be very irregular, and in some cases loses weight much faster than it does bulk, so that often the density of the material is decreased by firing. I shall endeavor to at least partially explain this phenomenon when speaking of the processes required for fusing porcelain inlays. It should be noted that the variation of formula in different porcelains may be entirely due to the different proportions in which the elements are mixed in Nature's laboratory, as the chemical formula for felspar varies greatly in different locations.

Porcelain teeth as made in England and America differ in the method of mixing and proportions. The English teeth are so arranged that the infusible material finely ground is held in suspension in the alkaline flux. The American teeth are a biscuit composed largely of infusible elements over and through which the alkaline flux is allowed to flow and penetrate. In the original manufacture of porcelain teeth the method now adopted by American manufacturers was used to some extent.

In the *Items of Interest* for 1901 it is stated that one Louis Beckers was the first to manufacture teeth. This is inaccurate, as the editor of that journal should have known. So far as I am able to find recorded accounts, it appears that the idea of making artificial teeth from porcelain was conceived by an apothecary, M. Duchatau, of St. Germain, France.[1] He applied to the porcelain workers of Paris in 1774, but the first attempts were not entirely a success because they failed to allow for the contraction in baking; several distinguished artists were consulted and also M. de Chemant, at that time dentist in Paris, who in 1808 published a pamphlet upon "The Advantage of the New Teeth in Entire Artificial Sets." A little later Dubois Foucou and M. Fonzi both published papers upon this subject; also Delabarre and Maurey wrote upon the subject of "Incorruptible Artificial Teeth."

In 1821 Joseph Audibran published his "Historical and Practical Treatise on Incorruptible Artificial Teeth," and gave as many as thirty or forty formulæ for their manufacture. The freedom with which these formulæ were given is in marked contrast to the attitude of some modern members of the profession who make proprietary articles of secret composition. These formulæ were all published in Fitch's "Dental Surgery," second edition, published

---

[1] Since writing the above the author has found a published claim that a M. Fouchard, of France, suggested and probably made artificial porcelain teeth as early as 1728.

in 1835. I do not know if the first edition, 1829, contained them or not.

Dr. A. A. Plantou was probably the first to introduce porcelain teeth in America, 1817, and later Samuel W. Stockton, Jones, White, and Dr. Wildman contributed to the perfecting of artificial teeth. These teeth were undoubtedly much higher fusing than the modern product, as they contained a much smaller proportion of fusible ingredients. A comparison of the fusing-point of the teeth upon the market at the present time may be of interest as showing the relative proportion of flux used by different manufacturers. These figures are in the Fahrenheit scale: Ash & Sons, 2264°; Sibley, 2408°; Dental Protective Supply Company, 2440°; Justi, 2440°; S. S. White, 2516°; Johnson & Lund, 2586°; Consolidated, 2624°; Dental Supply Company, 2624°.

I believe lime is added to the flux of the English teeth, which of itself will produce fusion at a lower heat than a potash flux. The silica in the S. S. White teeth, being ground to a less degree of fineness, gives them that appearance which a better refraction of light produces. That a porcelain tooth takes a polish easily after it has been ground does not necessarily prove that it is less porous; it may be due to the mechanical or crystalline structure of the harder tooth, the intractable crystals of silica not polishing away as rapidly as the softer flux. Any tooth of fine texture, like those made by the Consolidated or the Dental Supply Company, or the English teeth, will polish readily if first an Arkansas stone is used and then it be polished on the lathe with an impalpable silica powder.

As to inlays, which fad seems to be in the ascendancy just now with a specialty given to fads, there are two methods of making,—fitting a baked piece to the cavity and using the matrix of platinum or gold. The oldest one is to grind from an already baked porcelain a piece to fit the cavity; this method is first described by Dr. A. J. Volck in the *American Journal of Dental Science* for July, 1857, wherein he credits the suggestion to Professor Maynard, of Washington. These inlays were set in the cavity, leaving a narrow space around the edge, between the cavity wall and the porcelain, which was filled with gold. The walls of the cavity were to be left perpendicular, to keep the gold ring surrounding the inlay as narrow as possible. In a recent article treating upon the history of porcelain fillings, written by Dr. Bruck, of Germany, and translated by

Dr. Jenkins, it is stated that Dr. Murphy, of London, in 1837, used glass fused upon platinum as a filling. If so this is probably the origin of the low-fusing porcelain. The paper touches so lightly upon the history of inlay work in America that apparently the author is not properly posted upon the subject, or the translator, possibly, may have left out parts. Dr. Land, whom many consider to be the author of inlay work in which a matrix is used, is said to have fused pieces of artificial teeth in a platinum matrix in 1870. From this time on Dr. Land has been a persistent investigator along this line. Dr. Rollins, of Boston, has contributed much to the early knowledge of the matrix method. There are two distinct characteristics in the materials used with the matrix, high and low fusing. The higher the fusing, if the fused parts be perfectly homogeneous, the better. This is true porcelain; the low-fusing, properly speaking, is probably not a porcelain but a glass.

Of the high-fusing bodies, Close's, for continuous gum work, and a body made by Mr. G. H. Whiteley for Dr. Land, for inlay work, were the first to be put on the market. Mr. Whiteley was experimenting in this line as early as 1885; about 1889 he made up some high-fusing bodies for Dr. Land, and in 1898, at the instance of Dr. Joseph Head, he brought out and put on the market his high-fusing inlay body, which is still with us. The low-fusing materials, such as Herbst's, Ash & Sons' and Downie's, have come and gone, and the Jenkins body, which is always "just perfected" and constantly being changed to improve it, is the only one much used at present. The body made by Mr. Whiteley is still as much in use as any upon the market, though the S. S. White Company, Mr. Brewster, the Consolidated, and Ash & Sons are making a good high-fusing material also. The latter two fuse at a lower heat than the others, hence the colors burn out more readily. The Ash Company claim their high-fusing body requires more heat to bake than their teeth. This is a mistake, as a comparison of the fusing-points will show. High-fusing porcelain bodies for inlay work are of exactly the same materials as American teeth, but the proportions are changed, more alkaline flux being added. To a certain point this may not make much difference in the working qualities, or its durability in the fluids of the mouth. As you increase the amount of alkaline material the ease with which it combines with the metallic oxide becomes greater and the color becomes more fugitive. This process results in the formation of a gas or gases, and contributes largely to the bubbling or blistering of the inlay. This is why low-fusing body is more apt to lose color in fusing. In proportion as the fusing-point is raised the color becomes more stable. That body containing material ground to a greater degree of fineness, the proportions being the same, fuses at a lower heat, because, the infusible particles being extremely small, the flux blows over them when in a less plastic state, hence it is unnecessary to carry the heat to so high a point. The superior strength of the high-fusing material is due to the greater inherent strength of the intractable or non-fusible particles contained therein, and the greater the proportion of these elements the stronger the resulting substance, so long as there is enough fusible material to produce thorough homogeneous fusing of the matrix-forming flux.

The presence of silica in sufficient quantity and of a proper degree of coarseness gives stability to the mass during the fusing process and lessens shrinkage, making it easier to shape the inlay to conform to any particular outline. I doubt if a concavity which it is desirable to produce to harmonize with certain depressions in a tooth can be made with any low-fusing body except by grinding and reglazing. Under the microscope the higher-fusing bodies do not necessarily show a greater porosity than the low-fusing; it is simply different. In the first the air-spaces are irregular in shape, of varying sizes, and fewer in number, thus breaking up the rays of light better and giving a superior refraction. The edges when broken also show a clear-cut crystalline arrangement with sharp angles. In the latter the air-spaces are of small, even appearance, more numerous, similar in shape and size, and are better for the absorption of light, causing the peculiar dead color of the "porcelain enamel." Ordinary glass has about the same air-spaces, both in number, shape, and size, when examined under the microscope, as this latter material.

The appearance of the low-fusing body resembles glass fully as much as it does porcelain. It has neither the crystalline appearance nor the sharp angular edges where it is broken. I would suggest that the melting-point of gold, which is near 2000° F., is not necessarily the line of demarkation between high- and low-fusing body, but the melting-point of a good quality of felspar would be a better standard, and that it is not a question entirely of high or low fusing but of using the least amount of flux possible, in order to better retain the colors and to make it as little liable as need be to disintegration in the fluids of the mouth. I consider that it has never yet been proved that a body containing only those ingredients which are easily fused will not soon show signs of disintegration in the mouth. It should be borne in mind that kaolin is but the remains of disintegrated felspar which has become decomposed by long exposure to the atmosphere. In proportion as artificial flux is added to a body the tendency to unreliability increases, for if felspar be the base, as I understand it is in some low-fusing bodies, the whole mass is fusible and becomes glass, which will disintegrate sooner or later in moisture; if silica be present, though infusible itself, it may be rendered fusible by excess of alkali, and again you will produce glass, which as yet has not proved durable. It is not impossible that a glass may yet be produced which will meet the requirements obtaining in the mouth, but I shall doubt it until better evidence than is now at hand is produced.

In a high-fusing porcelain the strength is increased with the addition of infusible material. The crystalline silica may be said to act as a framework which would give strength and stability to the weaker material used as the flux or enamel. On the other hand, as the quantity of potash is increased the strength of the mass is proportionately diminished. If the silica is left too coarse, you get a rough or granular surface, which in some instances proves a weakness to the edge strength. Dr. Bruck claims that an analysis of the Jenkins powder shows it to be almost identical in composition with a high-fusing porcelain, the variation being very slight. This is not impossible, and yet the material might be a glass, for if all the ingredients were melted because of a change in proportions it would not be a porcelain, though containing the same elements. The claim that it is more dense has no relationship to its durability or strength.

Dr. Moffatt says, in a recent article in the *Dental Summary,* that porcelain enamels, so called, are nothing more nor less than a solution, when fused, of silex in glass.

The fusing-point of the different inlay materials used in the teeth will show, to some extent, the proportionate amount of alkaline solvent used. All these figures were given me by Mr. John F. Hammond, the maker of the Hammond electric furnace. The low-fusing bodies are as follows: Ash & Sons, 1544°; Jenkins, 1544°. The high-fusing: Whiteley's, 2210°; Consolidated, 2192°; S. S. White, 2300°; Close, 2300°; Brewster's body, 2210°; Brewster's enamel, 2084°; Moffatt's body, 2047°; Parker's, 2586°; Ash & Sons, 1904°.

In a recent number of the *Items of Interest* appears a paper in which, in the caution advocated in the beginning, there are some very reasonable and timely statements, but as one follows it along it is seen to contain much misleading sophistry. I will quote from it to show how reckless the advocates of "porcelain enamel" have become in their published statements. It is there stated that the feeling throughout the country that high-fusing porcelain is the most reliable has come about by the constant repetition of a few writers favoring high-fusing bodies.

It seems to me if he would put the proprietary name of "Porcelain Enamel" in place of "high-fusing" he would have described the case more accurately. Apparently a number of the enthusiastic supporters of porcelain enamel who have written or said much in its favor, with the exception of one or two like Dr. Gillett, have a personal interest in the commercial success of the manufacture of this powder. He also claims that the porcelain enamel is stronger. This hardly seems to be the fact. I am informed that to get its greatest strength it must be fused until it loses shape. The statement that the depth of the cavity increases the tendency to distort the matrix would need to be qualified in order to be accepted; but if this were so the gold matrix would bend much easier than the platinum after burnishing. And porcelain enamel draws into spherical form more readily than high-fusing body, and shrinks more. If annealed in the electric furnace platinum may be made thoroughly soft and pliable. The claim that as porcelain enamel will scratch glass it is therefore of great density, is entirely misleading. The density of a diamond is but 3.5, while mercury is 13.6, lead 11.3, platinum 21.4; this shows what density has to do with hardness. And finally, in order to advocate the porcelain enamel as superior in large cavities, the gentleman proceeds to juggle with the word inlay in order to give it a limited meaning, claiming that as it means a shallow body the high fusing supporters intended that material only for shallow cavities. Had the gentleman carefully read an article in his own journal antedating his own, he would have found where the earlier writer used the word enamel for shallow cavities and inlay for the deeper ones. It is my belief that in most cases where the condition of the patient and the attending circumstances permit, permanency is the most desirable thing in a filling. The high-fusing bodies have been in use for a long term of years, with little or no change in their composition; and have, when properly made and baked, and inserted in those places where indicated for æsthetic reasons, proved their utility as tooth-savers, as well as being the most harmonious and artistic imitation of tooth-structure that can be used.

The low-fusing body also originated some years ago, but so far, with the possible exception of the Porcelain Enamel, has proved a failure. This latter material, according to the statements of its author, has undergone numerous changes of formula, each change perfecting it, and yet experience apparently demonstrating the need of a further change. Under these circumstances, while I would not say that a low-fusing porcelain or glass cannot be produced that will prove durable, it seems the part of wisdom to use this material with a great deal of caution and only in those places where it can be carefully watched, until the time when the evidence in its favor is much less open to question than at present. It also seems to me, after a careful study of the written opinions of many disinterested writers who have used porcelain, that its use is limited under any circumstances, and that its physical qualities and methods of insertion are such as to make its accuracy of fit and durability very uncertain in complicated cases. It is also not impossible that as the fluids of the mouth vary in their composition in different individuals, so may the tendency to disintegration of porcelain inlays vary in different mouths.

Therefore it would seem that the question whether to use or not to use porcelain as a filling-material resolves itself into this form: Is the material durable in the conditions in which it is placed? And can the average individual, granting that a high-fusing material may be permanent, readily acquire the necessary skill and judgment to apply it with the greatest possible perfection, which is required to insure permanency, considering the limited call there must be for its application, until it has been more generally demonstrated that it has more qualities than the one of being æsthetic to recommend it to the careful, conscientious practitioner?

